# Supplementary material for: The role of non-axisymmetry of magnetic flux rope in constraining solar eruptions
Source: Nat Commun. 2021 May 12;12:2734. doi: 10.1038/s41467-021-23037-8 (PMC8115256; doi:10.1038/s41467-021-23037-8)
Supplement: Supplementary file 1 — Supplementary Information [file 41467_2021_23037_MOESM1_ESM.pdf]

Supplementary Information:

The role of non-axisymmetry of magnetic flux rope in constraining solar eruptions

Ze Zhong[1,2], **Yang Guo**[1,2✉], **and M. D. Ding**[1,2✉]

[1]School of Astronomy and Space Science, Nanjing University, Nanjing 210023, People's Republic of China

[2]Key Laboratory for Modern Astronomy and Astrophysics (Nanjing University), Ministry of Education, Nanjing 210023, People's Republic of China

email: guoyang@nju.edu.cn; dmd@nju.edu.cn

Contents:

Supplementary Figures 1–9

Supplementary Table 1

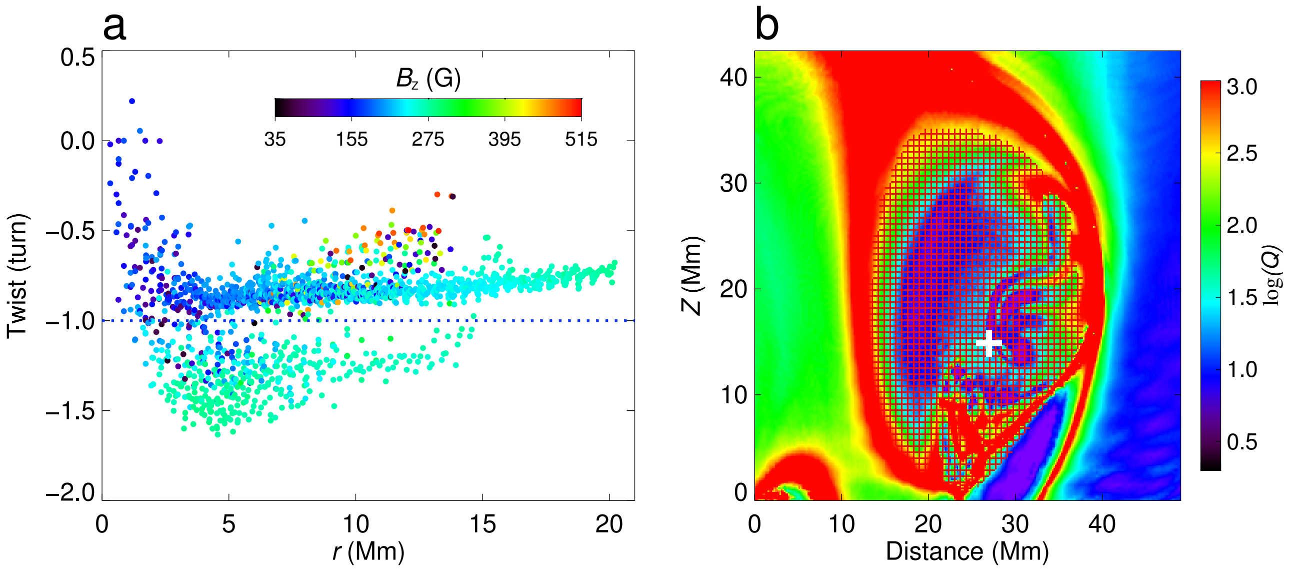

Supplementary Figure 1 | Magnetic properties of the initial flux rope. **a** The distribution of the twist number as a function of the distance to the MFR axis calculated from the initial reconstructed magnetic field. The strength of B_z measured in the positive polarity at the footpoints of the field lines is marked by different colors. **b** The position of the MFR axis labeled as a white plus symbol. Other field lines are labeled as red plus symbols, which are surrounded by the boundary of the MFR delineated by the QSLs shown on the transparent slice in Figure 2b.

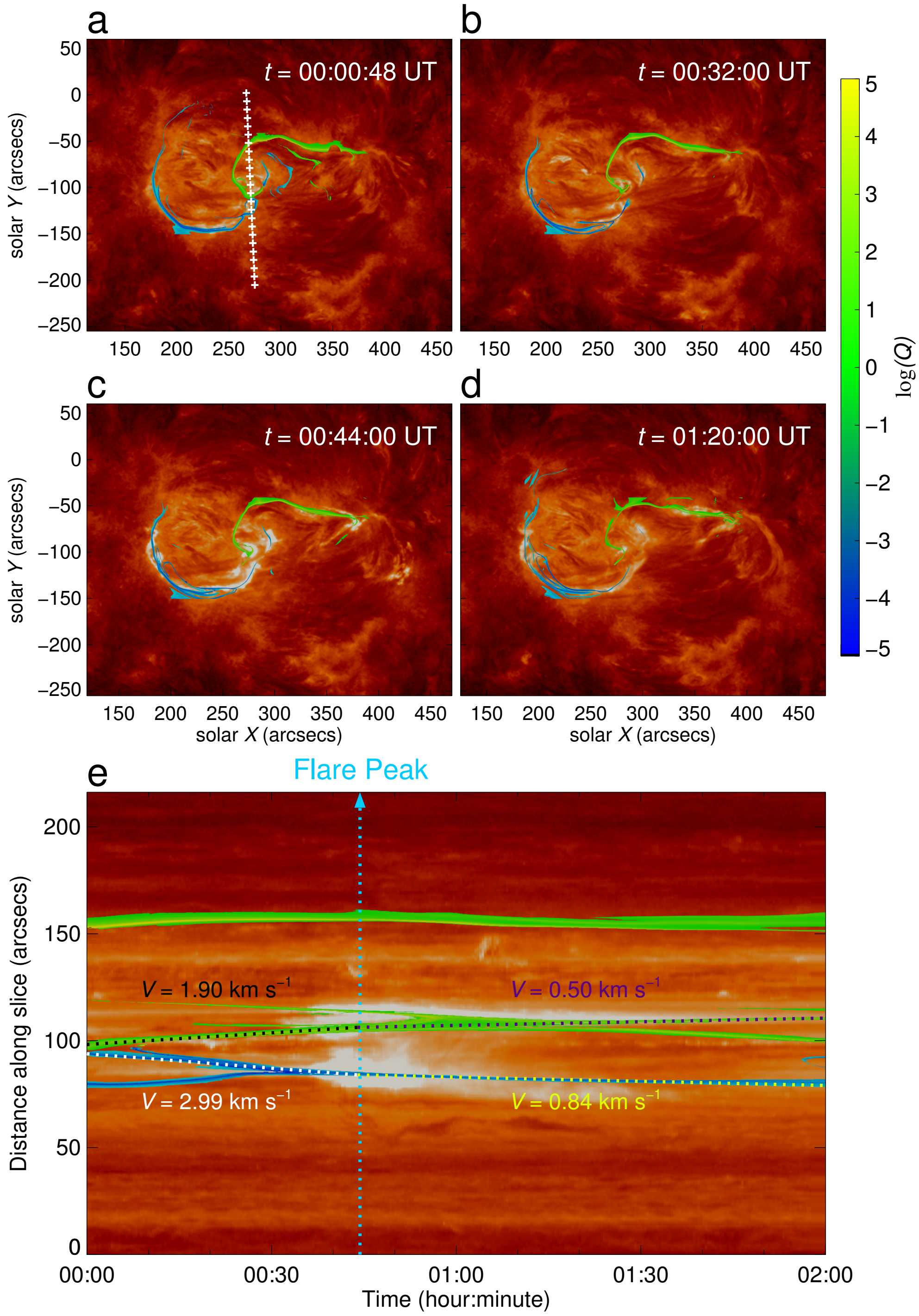

Supplementary Figure 2 | Comparison between the QSLs on the bottom and the extreme-ultraviolet emission in AIA 304 Å. **a–d** Time series of AIA 304 Å images at 00:00, 00:32, 00:44 and 01:20 UT on 30 January 2015, corresponding to the pre-flare, flare onset, flare peak and end times, respectively. The line of white plus symbols in panel **a** shows a slice crossing the flare ribbons. The QSLs with $|\log(Q)| > 3$, signed with positive and negative polarities, are overlaid on the 304 Å images. **e** Quantitative comparison between the signed QSLs with $|\log(Q)| > 3$ and the flare ribbons. The black and purple dotted lines show the separation motion of the flare ribbon in the positive polarity, with velocities being 1.9 km s^{-1} and 0.5 km s^{-1}, respectively. The white and yellow dotted lines show the motion of the flare ribbon in the negative polarity, with velocities being 2.99 km s^{-1} and 0.84 km s^{-1}, respectively. The cyan dotted line indicates the flare peak time at 00:44 UT.

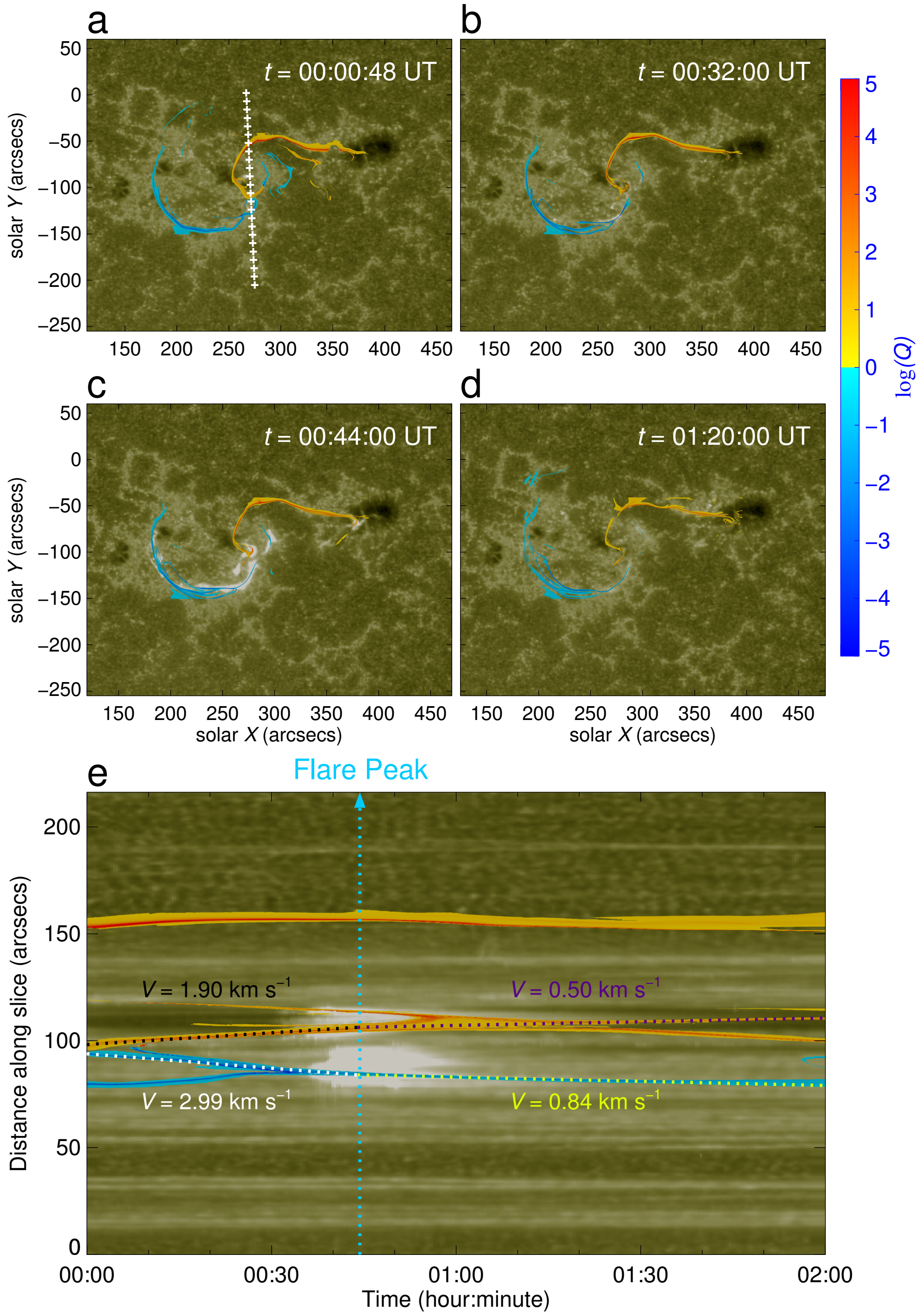

Supplementary Figure 3 | Comparison between the QSLs on the bottom and the extreme-ultraviolet emission in AIA 1600 Å. **a–d** Time series of AIA 1600 Å images at 00:00, 00:32, 00:44 and 01:20 UT on 30 January 2015, corresponding to the pre-flare, flare onset, flare peak and end times, respectively. The line of white plus symbols in panel **a** shows a slice crossing the flare ribbons. The QSLs with $|\log(Q)| > 3$, signed with positive and negative polarities, are overlaid on the 1600 Å images. **e** Quantitative comparison between the signed QSLs with $|\log(Q)| > 3$ and the flare ribbons. The black and purple dotted lines show the separation motion of the flare ribbon in the positive polarity, with velocities being 1.9 km s^{-1} and 0.5 km s^{-1}, respectively. The white and yellow dotted lines show the motion of the flare ribbon in the negative polarity, with velocities being 2.99 km s^{-1} and 0.84 km s^{-1}, respectively. The cyan dotted line indicates the flare peak time at 00:44 UT.

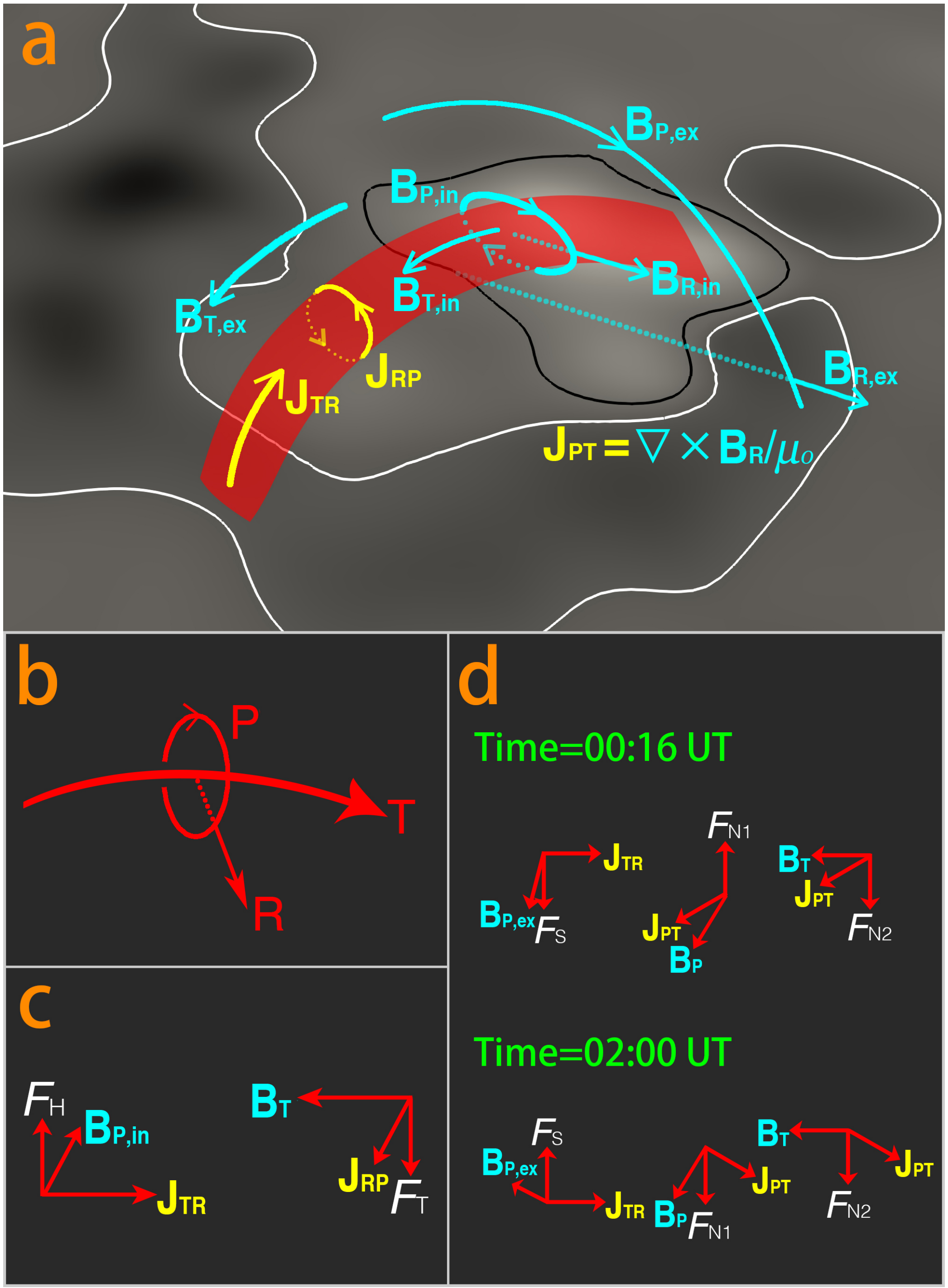

Supplementary Figure 4 | Schematic picture of magnetic fields, electric currents and Lorentz forces in the MFR. **a** The MFR depicted by the red semi-transparent surface. The magnetic field components ($\mathbf{B}_{\rm R}$, $\mathbf{B}_{\rm P}$, $\mathbf{B}_{\rm T}$) are shown in cyan while the electric current components ($\mathbf{J}_{\rm PT}$, $\mathbf{J}_{\rm TR}$, $\mathbf{J}_{\rm RP}$) are shown in yellow. The background image in black and white displays the SDO/HMI B_z component. **b** The local coordinate system used to define the three components. **c** Sketch map of the hoop and tension forces. **d** Sketch map of the strapping force and non-axisymmetry induced forces 1 and 2 at two moments before and after the flare onset, respectively.

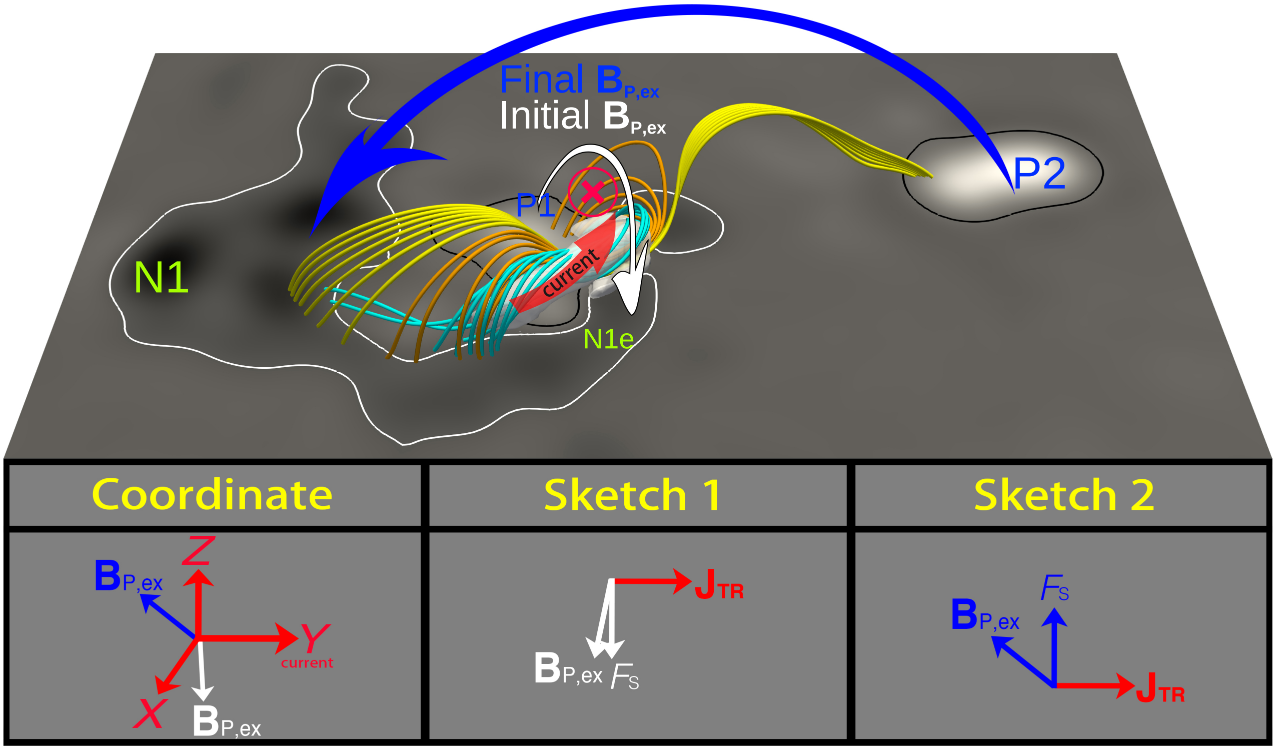

Supplementary Figure 5 | Sketch map of the strapping force. The yellow, orange and cyan lines represent the main body of the MFR. The white semi-transparent contour represents the electric current density that is larger than 17% of the maximum value in the whole domain. The direction of the electric current in the MFR points inwards. The long white and blue arrows represent the direction of the initial and final external poloidal fields, respectively. Also drawn at the bottom are the Cartesian coordinate system and two sketch maps of the strapping force corresponding to the initial and final fields. The fields associated with a downward force are shown in white, while the fields associated with an upward force are shown in blue. The background image shows the magnetogram with polarities labeled as N1, N1e, P1 and P2, overlaid by the white and black contours with contour levels of B_z being -50 G and 50 G, respectively.

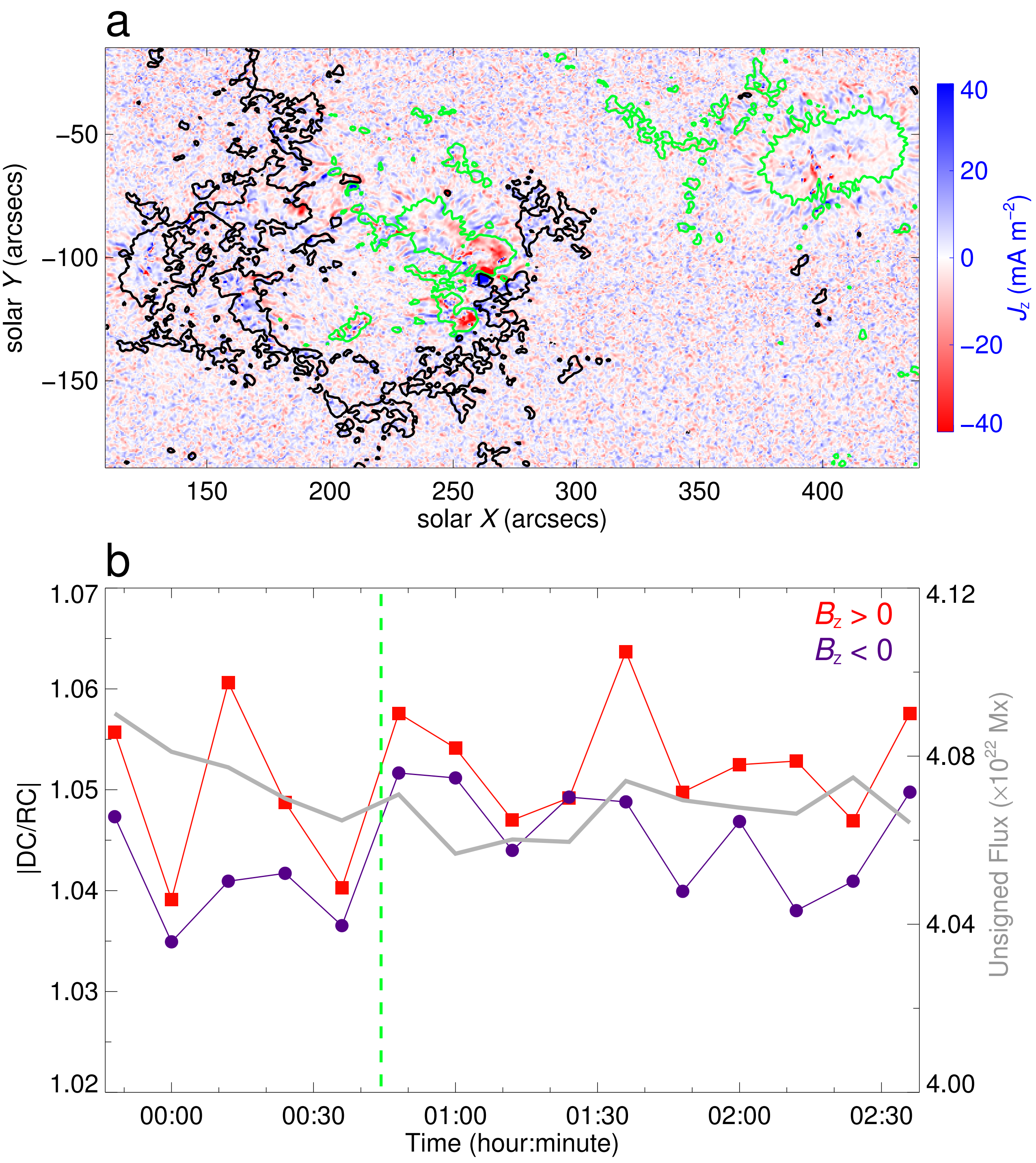

Supplementary Figure 6 | Temporal evolution of the vertical electric current density J_z. **a** The vertical electric current density J_z at 23:48 UT on 29 January 2015, which is derived from SDO/HMI vector magnetograms, overlaid by the black and green contours with contour levels of B_z being −300 G and 300 G, respectively. **b** The evolution of the unsigned magnetic flux (gray line) and the ratio of direct current to return current, |DC/RC|, in both positive (red line) and negative (purple line) polarities. The green dashed line represents the peak time of the M2.0 flare at 00:44 UT.

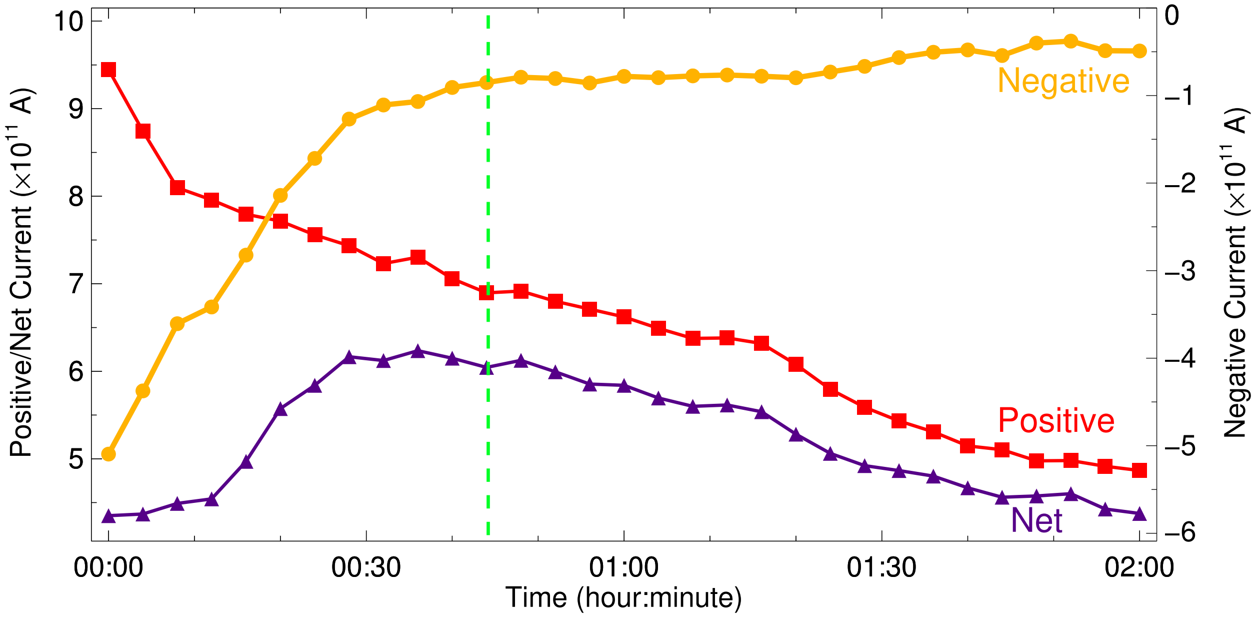

Supplementary Figure 7 | Temporal evolution of the axial electric currents of the MFR. The red, orange and purple lines represent the positive, negative and net electric currents, respectively. The green vertical line shows the peak time of the M2.0 flare at 00:44 UT.

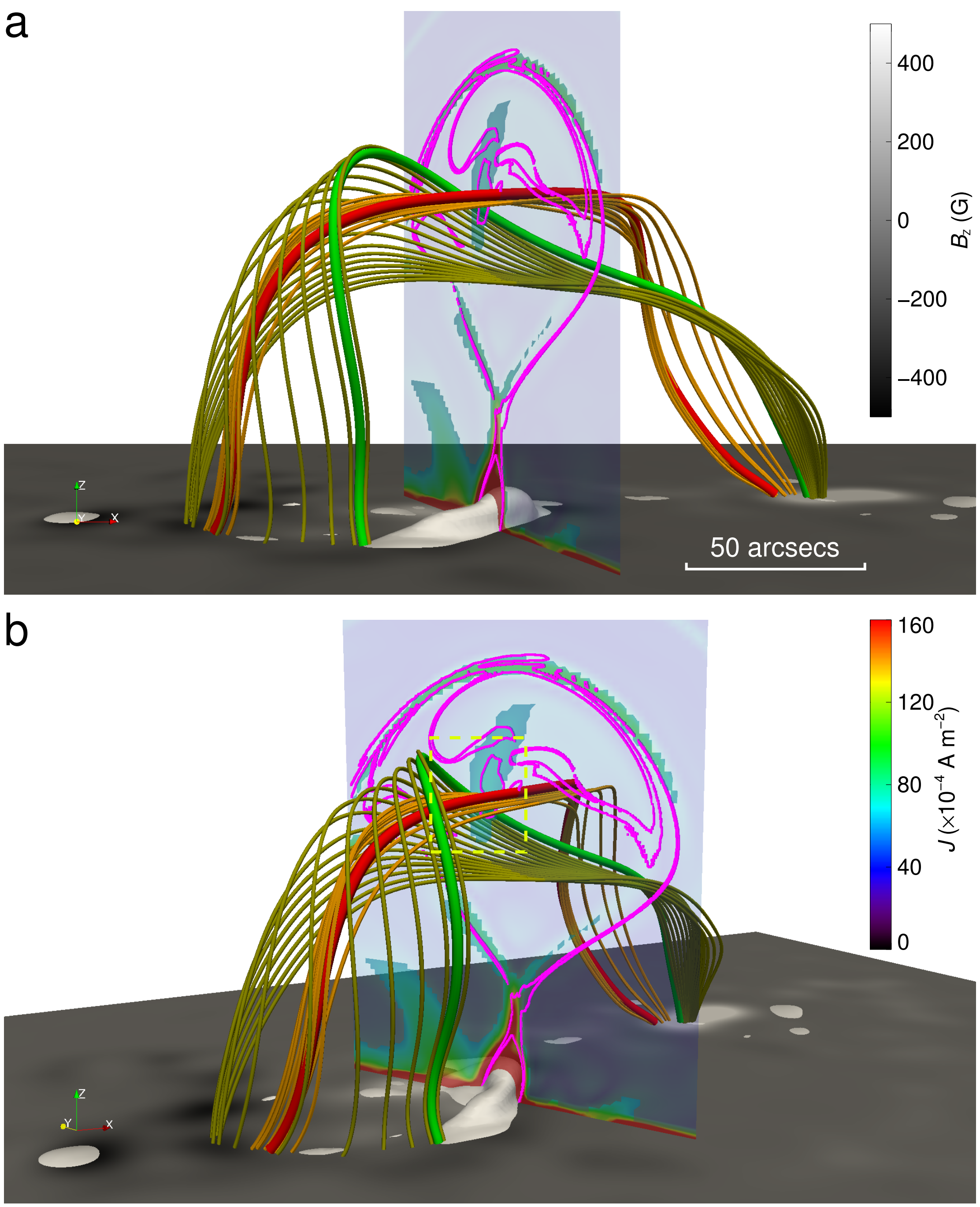

Supplementary Figure 8 | Selected magnetic field lines and electric current density at 01:20 UT showing a possible internal reconnection. **a** A side view along the Y-axis. The vertical transparent slice displays the electric current density depicted by a high Q value of $\log(Q) > 3$ (in pink). The red and green lines represent a configuration similar to the bald-patch separatrix surface. The orange lines represent a group of lines close to the red line, while the olive lines are a group close to the green line. The white isosurface represents the electric current density larger than 32.9% of the maximum value in the whole domain. The background shows the distribution of the vertical magnetic field component, B_z. **b** A side view along the green field line at its apex. The yellow dashed box depicts a region where the green and red field lines meet each other.

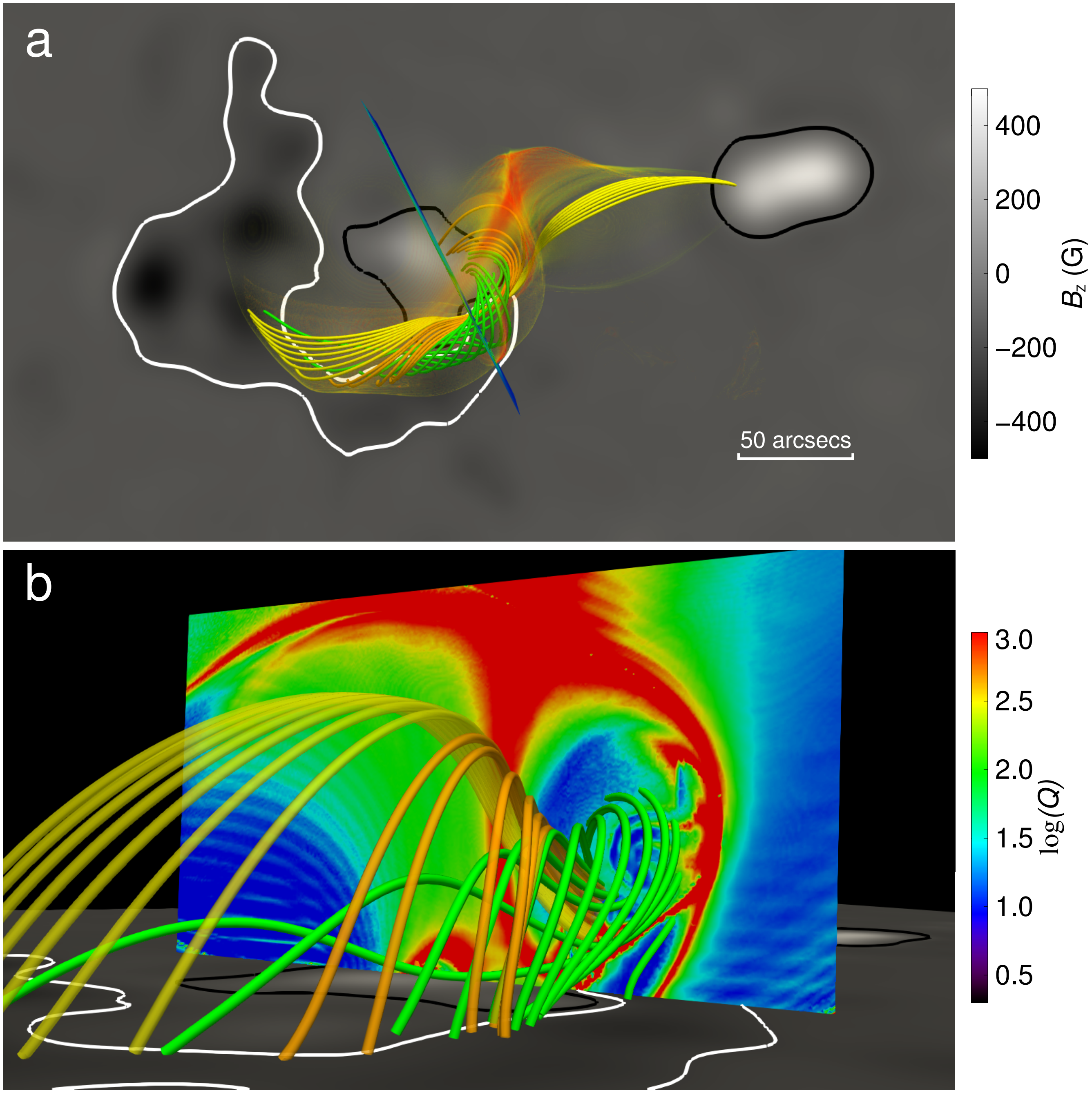

Supplementary Figure 9 | Overview of the MFR structure shown with magnetic field lines and the Q value. The 3D semi-transparent yellow boundary represents a high Q value of $\log(Q) > 3$. **a** Selected field lines of the MFR. The yellow and orange lines represent the bifurcated and unbifurcated parts of the MFR, respectively. The green lines show the main body of the MFR. The background image in black and white displays the SDO/HMI B_z component. **b** An 2D slice displaying the boundary of the MFR depicted by a high Q value (in red). The slice and the field lines are the same as that in panel **a** but with a different viewing angle.

Supplementary Table 1: Different components of the magnetic field, electric current and Lorentz force

Quantity	Symbol[a,b]	Expression
External radial magnetic field	$\mathbf{B}_{\mathrm{R,ex}}$	...
Rope radial magnetic field	$\mathbf{B}_{\mathrm{R,in}}$	...
External poloidal magnetic field	$\mathbf{B}_{\mathrm{P,ex}}$	...
Rope poloidal magnetic field	$\mathbf{B}_{\mathrm{P,in}}$	...
External toroidal magnetic field	$\mathbf{B}_{\mathrm{T,ex}}$	...
Rope toroidal magnetic field	$\mathbf{B}_{\mathrm{T,in}}$	...
Total radial magnetic field	$\mathbf{B}_{\mathrm{R}}$	$\mathbf{B}_{\mathrm{R,ex}} + \mathbf{B}_{\mathrm{R,in}}$
Total poloidal magnetic field	$\mathbf{B}_{\mathrm{P}}$	$\mathbf{B}_{\mathrm{P,ex}} + \mathbf{B}_{\mathrm{P,in}}$
Total toroidal magnetic field	$\mathbf{B}_{\mathrm{T}}$	$\mathbf{B}_{\mathrm{T,ex}} + \mathbf{B}_{\mathrm{T,in}}$
Radial field induced current	$\mathbf{J}_{\mathrm{PT}}$	$\nabla \times \mathbf{B}_{\mathrm{R}}/\mu_0$
Poloidal field induced current	$\mathbf{J}_{\mathrm{TR}}$	$\nabla \times \mathbf{B}_{\mathrm{P}}/\mu_0$
Toroidal field induced current	$\mathbf{J}_{\mathrm{RP}}$	$\nabla \times \mathbf{B}_{\mathrm{T}}/\mu_0$
Hoop force density	F_{H}	$\mathbf{e}_{\mathrm{z}} \cdot (\mathbf{J}_{\mathrm{TR}} \times \mathbf{B}_{\mathrm{P,in}})$
Strapping force density	F_{S}	$\mathbf{e}_{\mathrm{z}} \cdot (\mathbf{J}_{\mathrm{TR}} \times \mathbf{B}_{\mathrm{P,ex}})$
Tension force density	F_{T}	$\mathbf{e}_{\mathrm{z}} \cdot (\mathbf{J}_{\mathrm{RP}} \times \mathbf{B}_{\mathrm{T}})$
Non-axisymmetry induced force 1	F_{N1}	$\mathbf{e}_{\mathrm{z}} \cdot (\mathbf{J}_{\mathrm{PT}} \times \mathbf{B}_{\mathrm{P}})$
Non-axisymmetry induced force 2	F_{N2}	$\mathbf{e}_{\mathrm{z}} \cdot (\mathbf{J}_{\mathrm{PT}} \times \mathbf{B}_{\mathrm{T}})$

[a] The subscript "ex" refers to the field components of the potential field, while the subscript "in" refers to the field components that are obtained after subtracting the "ex" components from the total field.

[b] Notations for the total poloidal magnetic field, $\mathbf{B}_{\mathrm{P}}$, the total toroidal magnetic field, $\mathbf{B}_{\mathrm{T}}$, and their decompositions are the same as that used by Myers et al. (2015).

References

1. Myers, C. E. *et al.* A dynamic magnetic tension force as the cause of failed solar eruptions. *Nature* **528**, 526–529 (2015).
