# Supplementary material for: The role of non-axisymmetry of magnetic flux rope in constraining solar eruptions
Source: Nat Commun. 2021 May 12;12:2734. doi: 10.1038/s41467-021-23037-8 (PMC8115256; doi:10.1038/s41467-021-23037-8)
Supplement: Supplementary file 3 — Description of Additional Supplementary Files [file 41467_2021_23037_MOESM3_ESM.pdf]

Description of Additional Supplementary Files

Supplementary Movie 1:

Temporal evolution of the magnetic field lines and the electric current density. The movie displays a central part of the full computation domain, namely, X = [171″, 382″] and Y = [$-$210″, 105″] in the X–Y plane. The vertical transparent slice displays the electric current density. The cyan, yellow and orange lines have the same meaning as that in Figure 2b, only that more field lines are drawn here. The olive lines refer to field lines connecting the negative polarity N1 and the positive polarity P2 at the first moment. The white isosurface represents the electric current density larger than 32.9% of the maximum value in the whole domain. The background shows the distribution of the vertical magnetic field component, B_z.

Supplementary Movie 2:

Temporal evolution of the SDO/AIA 304 Å images and the signed QSLs with $|\log(Q)| > 3$.

Supplementary Movie 3:

Temporal evolution of the QSLs (left) and the Lorentz force, L_z (right). The background at the bottom shows the distribution of the vertical magnetic field component, B_z. The yellow and orange semi-transparent isosurfaces represent the electric current density larger than 21.9% and 32.9% of the maximum value in the whole domain, respectively.
